# Prosthetic Materials Used for Implant-Supported Restorations and Their Biochemical Oral Interactions: A Narrative Review

**DOI:** 10.3390/ma15031016

**Published:** 2022-01-28

**Authors:** Roxana Nicoleta Ionescu, Alexandra Ripszky Totan, Marina Meleșcanu Imre, Ana Maria Cristina Țâncu, Mihaela Pantea, Mihai Butucescu, Alexandru Titus Farcașiu

**Affiliations:** 1 Faculty of Dental Medicine, “Carol Davila” University of Medicine and Pharmacy, 17-23 Plevnei Street, 020021 Bucharest, Romania; roxana-nicoleta.ionescu@drd.umfcd.ro; 2 The Interdisciplinary Center for Dental Research and Development, Department of Biochemistry, Faculty of Dental Medicine, “Carol Davila” University of Medicine and Pharmacy, 17-23 Plevnei Street, 020021 Bucharest, Romania; alexandra.totan@umfcd.ro; 3 Department of Complete Denture, Faculty of Dental Medicine, “Carol Davila” University of Medicine and Pharmacy, 17-23 Plevnei Street, 020221 Bucharest, Romania; marina.imre@umfcd.ro; 4 Department of Fixed Prosthodontics and Occlusology, Faculty of Dental Medicine, “Carol Davila” University of Medicine and Pharmacy, 17-23 Plevnei Street, 020221 Bucharest, Romania; 5 Department of Operative Dentistry, Faculty of Dental Medicine, “Carol Davila” University of Medicine and Pharmacy, 17-23 Plevnei Street, 020221 Bucharest, Romania; mihai.butucescu@umfcd.ro; 6 Department of Removable Prosthodontics, Faculty of Dental Medicine, “Carol Davila” University of Medicine and Pharmacy, 17-23 Plevnei Street, 020221 Bucharest, Romania; alexandru.farcasiu@umfcd.ro

**Keywords:** prosthetic dental materials, implant-supported restorations, biochemical interactions

## Abstract

The purpose of this study is to outline relevant elements regarding the biochemical interactions between prosthetic materials used for obtaining implant-supported restorations and the oral environment. Implant-supported prostheses have seen unprecedented development in recent years, benefiting from the emergence of both new prosthetic materials (with increased biocompatibility and very good mechanical behavior), and computerized manufacturing technologies, which offer predictability, accuracy, and reproducibility. On the other hand, the quality of conventional materials for obtaining implant-supported prostheses is acknowledged, as they have already proven their clinical performance. The properties of PMMA (poly (methyl methacrylate))—which is a representative interim material frequently used in prosthodontics—and of PEEK (polyether ether ketone)—a biomaterial which is placed on the border between interim and final prosthetic use—are highlighted in order to illustrate the complex way these materials interact with the oral environment. In regard to definitive prosthetic materials used for obtaining implant-supported prostheses, emphasis is placed on zirconia-based ceramics. Zirconia exhibits several distinctive advantages (excellent aesthetics, good mechanical behavior, biocompatibility), through which its clinical applicability has become increasingly wide. Zirconia’s interaction with the oral environment (fibroblasts, osteoblasts, dental pulp cells, macrophages) is presented in a relevant synthesis, thus revealing its good biocompatibility.

## 1. Introduction

The introduction of endosseous dental implants into dental practice has opened new perspectives in the prosthetic treatment of partially and completely edentulous patients. Today, it is widely considered that the use of endosseous dental implants has become a routine clinical procedure; the favorable long-term outcomes of dental implant therapy have been acknowledged in the scientific literature, the reported survival rate of dental implants being more than 90% after a follow-up period of at least 5 years [1].

Several types of prosthetic designs and materials are available nowadays for implant-supported fixed prostheses, depending on their type: interim (provisional) or definitive (final) [2,3]. The survival rate of implant-supported fixed prostheses is influenced by several factors, such as the prosthesis type, prosthesis retention mechanism, design of the supporting framework, prosthesis manufacturing process, or the length and type of implant [4,5,6]. The selection of the interim or final prosthetic materials for obtaining the implant-supported restorations is an additional factor to be considered along with the aforementioned variables, as it might play a role in influencing the success-rate of this specific prosthetic treatment [7]. Through the advancements of dentistry, the use of metal–acrylic restorations on implants was replaced with the use of metal–ceramic restorations, in order to overcome the drawbacks of metal–acrylic restorations [8]. New materials are in use nowadays for obtaining these prostheses, such as monolithic zirconia, ceramic-veneered zirconia, ceramic-veneered titanium, lithium disilicate, hybrid ceramics, milled PMMA (poly (methyl methacrylate)), PEEK (polyether ether ketone), or 3D-printed resins, all of which present revered biological and mechanical properties.

As conceptually defined in 1980, the term biocompatibility “refers to the ability of a material to perform with an appropriate host response in a specific application”, being one of the most critical factor that controls the success of biomaterials [9]. This feature is not a property of a biomaterial, even if controlled by the nature of it, but a characteristic of a material–tissue system, influenced by many other complex factors. The mechanism of interactions between biomaterials and the human body, and the consequences of these interactions, are to be taken into consideration; these aspects are of great importance in the domain of prosthetic materials used for obtaining implant-supported prosthesis [9].

Multiple studies on aspects that influence the survival rate of implants are found in scientific literature, but relatively few studies are available on the interactions of prosthetic materials with the oral environment [10]. Long-term complications of implant-supported fixed dental prostheses can be technical and biological [11]. The technical complications consist of infrastructure fracture, fracture of the veneering ceramics, screw loosening, or loss of retention due to prosthesis de-cementation [11]. On the other hand, peri-implantitis is one of the most common biological complications related to dental implant therapy. Peri-implantitis has been defined as an inflammatory disease induced by bacteria; its unfavorable evolution can lead to progressive loss of supporting bone. Probing pocket depth (PPD) > 5 mm under light force, profuse bleeding on probing (BOP), and suppuration on probing (SOP) were determined as clinical parameters associated with peri-implantitis [12]. All of the above-mentioned complications could be associated with the materials’ biochemical interaction with the oral environment.

Due to the variable design of the scientific studies regarding the materials used for obtaining implant-supported fixed prostheses, it is quite challenging to assess the influence of diverse prosthetic materials on the oral environment; moreover, the impact of prosthodontic material selection on the endosseous implant survival-rate is not yet very clear.

The aim of this paper is to present the biochemical interactions of interim and definitive prosthetic materials used for obtaining implant-supported fixed prostheses within the oral environment, highlighting the possible biological complications associated with these materials. Additionally, this paper aims to present aspects related to the survival-rate of implant-supported fixed prostheses, in terms of materials used in the manufacturing of prostheses.

## 2. Interim Prosthetic Materials Used for Obtaining Implant-Supported Prosthesis

### 2.1. The Role of Interim Prosthesis in Oral Implant Therapy

Obtaining an implant-supported fixed prosthesis includes, prior to the delivery of the final implant restorations, the use of an interim prosthesis, in order to evaluate the aesthetics, phonatory function, and masticatory function while preserving and/or enhancing the condition of the peri-implant and gingival tissues. The interim prosthesis is also a very useful communication tool between members of the dental team [13,14]. In order to make the best choice when selecting a specific restorative material for obtaining interim implant-supported prosthesis, the dental practitioner should be familiar with materials’ properties; apart from their mechanical properties, interim prostheses should exhibit a minimally invasive interaction with the oral environment through which favorable equilibrium is maintained. The health of the gingiva located near an interim crown is influenced by several factors, among which the material’s characteristics have a great significance.

### 2.2. Materials and Techniques Used for Fabrication of Oral Interim Implant-Supported Prosthesis

The interim prosthetic restorations can be obtained by using conventional direct techniques (chairside fabrication), indirect techniques (dental laboratory fabrication), or indirect–direct techniques. Modern techniques for obtaining interim prostheses include the use of CAD/CAM (computer-aided design/computer-aided manufacturing) technologies, such as the subtractive method (milling) or the additive one (3D printing) [15]. Interim prosthetic restorations are obtained from different types of dental materials, including conventional ones (based on monomethacrylate or acrylic resins and methacrylates or bis-acryl/composite resins) and modern ones such as milled PMMA (poly (methyl methacrylate)) or 3D-printed resins. CAD/CAM provisional crowns (made of PMMA (poly (methyl methacrylate)) and PEEK (polyether ether ketone) showed superior marginal fit and better strength than direct provisional crowns [16,17]. In the same line, Rayyan et al. [18] showed that CAD/CAM PMMA (poly (methyl methacrylate)) blocks offer, due to their optimum manufacturing conditions, better mechanical properties and chemical stability than those that are manually fabricated [19,20,21]. The valuable properties of milled PMMA (poly (methyl methacrylate)) represent an argument for using them for long-term interim prostheses, when strength, optimal biocompatibility, and color stability are required. Additionally, the improved fit of the milled CAD/CAM interim tooth-supported prostheses, should lower the risk of bacterial contamination of the tooth and prevent damage to the pulp from excessive temperature changes [22].

One of the most important challenges of modern scientific research in dentistry is to improve the biocompatibility and biomechanical properties of dental materials, by exploring the molecular landscape of material–oral tissue interactions [23]. There is an increasing trend of using metal-free materials in the oral environment in order to avoid ion release and corrosion problems. This trend has led to the development of chemically inert polymers [23]. Polymers are macromolecules synthesized from smaller molecules, monomers, which can form linear or racemic chains [24]. Most polymers have lower elastic moduli and ensure greater elongation to fracture, compared to other types of biomaterials. Polymer macromolecules have great resistance to biodegradation [23]. Two of the most used polymeric biomaterials in dentistry are PMMA (poly (methyl methacrylate)) and polyaryletherketone (PAEK) [25].

### 2.3. PMMA (Poly (methyl methacrylate))—An Acknowledged Material Used for Obtaining Oral Interim Implant-Supported Prosthesis with Its Performance Still Being under Evaluation

**Figure 1:**
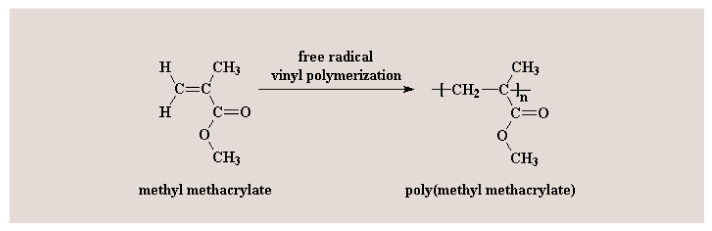
Methyl methacrylate polymerization reaction.

PMMA (poly (methyl methacrylate)) is an acrylic-based self-polymerizing resin [26]. In dentistry, the PMMA (poly (methyl methacrylate)) polymer is prepared using liquid methyl methacrylate (MMA) monomer and a pre-polymerized PMMA (poly (methyl methacrylate)) powder [26]. The methyl methacrylate polymerization reaction is shown in Figure 1. Practically, the methyl methacrylate monomers (MMA) polymerization reaction is incomplete. Consequently, unpolymerized MMA (methyl methacrylate) monomers can be released into saliva and interact with the oral tissues [27,28]. Patient saliva analyses after dental restorative procedures, including completed polymerization, confirmed the presence of MMA (methyl methacrylate) monomer in saliva [29,30]. In vitro elution studies also revealed the MMA (methyl methacrylate) monomers solubilization during the polymerization reaction [31]. It has been shown that in the clinical situations when PMMA (poly (methyl methacrylate)) restorations are placed on the prepared teeth (such as in the case of interim crowns), unreacted MMA monomer was able to diffuse through dentin via dentinal tubules, reaching the pulp tissue, due to their small size [32,33]. MMA (methyl methacrylate) monomer triggers complex biological effects in the dental pulp cells [32,33,34,35]. In the same context, Galler et al. have pointed out that the unpolymerized MMA (methyl methacrylate) monomer released from the resin induced the disruption of specific odontoblast functions: dentin sialoprotein gene activity, alkaline phosphatase activity and, consequently, and the matrix mineralizing capability [36]. Moreover, experimental data revealed that the resin monomer significantly affected the differentiation of pulpal stem cells and the mineralization processes, triggering the disruption of the physiological dentine repair process [37,38,39].

Previous studies have highlighted the adverse effects and toxicity of PMMA (poly (methyl methacrylate))-based dental materials, at both the tissue and cellular levels, although systemic toxicity is rarely reported [35,40,41,42,43]. Studies have shown that local adverse effects in tissues next to devices made from PMMA (poly (methyl methacrylate)) may include fibrosis, histiocytosis, and necrosis [35,40,41,42,43].

At the cellular level, the unreacted MMA (methyl methacrylate) monomers have been involved in the disruption of vital cellular events such as differentiation, proliferation, and apoptosis. For instance, Granchi et al. have pointed out that the PMMA (poly (methyl methacrylate)) resin extracts inhibited osteoblast proliferation [44]. Moreover, Ciapetti et al. reported that the PMMA (poly (methyl methacrylate))-based materials have induced apoptosis and also the cellular necrosis of osteoblastic cell lines, probably via unreacted MMA (methyl methacrylate) monomer [45]. MMA (methyl methacrylate) effects on gene mutation and cell death have been shown to be extensively demonstrated in fibroblasts or fibroblastic cells [46].

Ratanasathien et al. concluded that the mechanisms of PMMA (poly (methyl methacrylate)) toxic effects might involve direct toxicity of the released or residual MMA monomer and/or the oxidative stress (OS) generated by the free radicals released by the polymerization initiator and the resin per se [47]. Studies conducted on permanent cell lines or primary cultured cells from the gingiva, periodontal ligament, and dental pulp revealed that MMA (methyl methacrylate) monomer and PMMA (poly (methyl methacrylate)) induced cytotoxic effects via the apoptotic cascade [46,48,49]. It has also been reported that MMA (methyl methacrylate) monomer and PMMA (poly (methyl methacrylate)) exposure induced genotoxic effects and cell-cycle delays [46,48,49]. Krifka et al. also highlighted that the molecular mechanisms behind the PMMA (poly (methyl methacrylate)) toxicity involves OS (oxidative stress) initiation and evolution [48]. Moreover, experimental results revealed that cell exposure to MMA monomer decreased the glutathione (GSH) levels and stimulated reactive oxygen species (ROS) generation [48,50]. Intracellular ROS accumulation, beyond the concentration that ensures their roles in cellular signaling, represents the main step to oxidative stress and should be considered the first ample molecular response to environmental attacks, including the interactions with resin monomers, such as MMA monomers [50,51]. Jiao et al. reported increased levels of ROS (reactive oxygen species) after a short exposure of cultured cells to PMMA (poly (methyl methacrylate)) [50,51]. It has been intensively highlighted that OS (oxidative stress), once installed, triggers the disruption and damage of vital cellular signaling pathways and functions, due to different degrees of protein and lipid macromolecules peroxidation, and DNA oxidative damage, as well as [50]. Recently, Jiao et al. reported significant activity changes of glutathione peroxidase (GPx), superoxide dismutase (SOD), and catalase (CAT), and increased levels of malondialdehyde (a lipid peroxidation product), in cells that were exposed to MMA monomer [51]. It is important to note that the expression of the antioxidative enzymes, such as GPx, SOD, CAT, is regulated by the nuclear factor erythroid 2-related factor 2 (Nrf2) [52,53,54]. Remaining in this context, recently, it has been demonstrated that the activation of Nrf2-controled cellular antioxidative equipment reduced the resin monomer-induced OS (oxidative stress) and ensured cell viability [52,53,54]. The findings of Zhang et al. revealed that Nrf2 pharmacological activation by tert-Butylhydroquinone/2-tert-butylbenzene-1,4-diol tert-butylhydroquinone protected the PMMA (poly (methyl methacrylate)) exposed cells from resin-induced apoptosis [55]. These results open new ways to effective therapeutic targets, important to ensure cells’ adaptation under the MMA monomer-induced OS conditions [55]. The extremely complex redox relationship between the antioxidant response and autophagy, unfolding through the p62/Keap1/Nrf2 molecular pathway, might indicate the subtle role of autophagy in the PMMA (poly (methyl methacrylate))-induced OS (oxidative stress) landscape [56].

However, in the PMMA (poly (methyl methacrylate))-induced OS (oxidative stress) context, the complex autophagy–apoptosis relationship, in which ROS (reactive oxygen species) are very important pawns, is yet to be clarified. It has been highlighted that the autophagy machinery is interconnected in a complex way, with apoptosis, in both physiological and pathological conditions [57,58]. Becker et al. have reported that methacrylic acid-based compounds could activate the autophagy cascade [59]. Moreover, it has been revealed that PMMA (poly (methyl methacrylate)) microcapsules induced autophagy in different cell types [60,61].

Autophagy, a highly controlled pathway which is responsible for the degradation of intracellular components, is also involved in the cellular response to stress, including OS (oxidative stress) [56]. Wen et al. reported that intracellular ROS play important and complex roles, as signaling molecules, in autophagy regulation [62]. It has also been pointed out that ROS are involved in the autophagosomes stabilization in stressful conditions, such as nutrient deprivation, hypoxia, and ischemia reperfusion injury. At a physiological level, ROS (reactive oxygen species) play key roles in regulating the molecular pathways involved in cellular adaption to stress and survival. However, redox equilibrium disruption by excessive ROS (reactive oxygen species) generation induced irreversible cellular damage, thereby accelerating the autophagy cascade and/or apoptotic death [62]. One of the main molecular events involved in intracellular ROS (reactive oxygen species) accumulation is the depolarization of mitochondrial membranes [63]. The appearance of mitochondrial membrane lesions has severe consequences, including increased ROS (reactive oxygen species) generation, decreased ATP synthesis, and the redistribution of pro-apoptotic mitochondrial factors [63].

Autophagy involves the autophagosomes assembly [64,65]. The autophagosomes are double-membraned vesicles that are able to sequester cytoplasm and organelle residues. After the autophagosomes assembly is completed, these vesicles subsequently fuse with the lysosomes and form the autolysosomes, in order to finish the degradation of residues [64,65]. Autophagy, a very complex molecular machinery, should be regarded as a double-edged sword, playing either the role of cell survival mechanism or that of cell death promoter, depending on the environmentally stressful conditions and on the cell types [66,67]. As a pro-survival mechanism, autophagy becomes an energy source due to bulk degradation. However, in specific conditions, this molecular pathway interacts in complex ways with the apoptotic pathway and becomes a cellular death promotor [66,67,68,69]. It still remains to be clarified whether the complex molecular events that involve autophagy occur after cell exposure to PMMA (poly (methyl methacrylate)) and/or MMA monomer. The way autophagy is able to control resin monomer-induced toxicity in dental mesenchymal cells remains in question.

As previously highlighted, MMA monomer and PMMA (poly (methyl methacrylate)) cause cytotoxicity via complex molecular events, in which the key roles are attributed to ROS generation, OS, autophagy, and, finally, possibly to apoptosis [48]. Based on the experimental results that highlighted the key role played by OS in the molecular mechanisms of PMMA (poly (methyl methacrylate)) material toxicity, a new challenge is represented by studying the effects of antioxidants in the resin exposure context. ROS scavenging molecules and antioxidants such as *N*-acetyl cysteine (NAC) have been proposed to be used for the protection of PMMA (poly (methyl methacrylate))/MMA exposed cells [51,70,71,72]. In order to prevent MMA-induced oxidative stress and, consequently, apoptotic cell death, in human dental pulp cells, Jiao et al. have studied the efficacity of NAC (*N*-acetyl cysteine), a cell-permeable compound [51]. NAC (*N*-acetyl cysteine) is a cysteine derivative and represents a key glutathione (GSH) precursor [51]. Rushworth et al. revealed that NAC (*N*-acetyl cysteine) acts as a direct ROS scavenger but also accelerates the intracellular GSH redox-cycle [73]. Jiao et al. have pointed out that high mass fractions of NAC in the PMMA (poly (methyl methacrylate)) resin triggered alterations in the microhardness, surface roughness and flexural strength, limiting NAC concentration to 0.15 wt.%, PMMA (poly (methyl methacrylate)) resin biocompatibility was remarkably improved, without any significant negative consequences on the mechanical properties [72]. There are also studies that mentioned NAC incorporation into the PMMA (poly (methyl methacrylate)) resin materials as a method for improving the resin’s biocompatibility [74,75]. First, it was believed that NAC (*N*-acetyl cysteine) molecular protective effects against resin monomer-induced cytotoxicity involved direct ROS scavenging and GSH formation [51,55]. Moreover, it has also been reported that NAC molecules were able to reduce the availability of monomers from dental resin monomers by direct chemical reaction with their methacrylic group [72,76,77]. However, Zhang et al. highlighted that high NAC (*N*-acetyl cysteine) concentrations reduced drastically the intracellular ROS levels below the critical physiological levels, vital for ROS roles as signals in essential molecular pathways involved in the complex regulation of cell vitality and proliferation [55]. Therefore, the incorporation of NAC (*N*-acetyl cysteine), but only in appropriate proportions, might be regarded as an effective strategy to obtain biocompatible and clinically reliable PMMA (poly (methyl methacrylate))-based dental resins [72].

PMMA (poly (methyl methacrylate))-based interim prosthetic materials interact in a very complex way with the oral environment; thus, diverse considerations regarding their possible adverse oral effects were presented above. However, PMMA (poly (methyl methacrylate)) has already proved its clinical performance in the context of obtaining favorable final prosthetic results, being one of the most frequently used materials for obtaining reliable interim dental prostheses [78].

### 2.4. PEEK (Polyether Ether Ketone)—A High Performance Polymer for Interim & Definitive Use in Prosthodontics

Metal–ceramic implant-supported prostheses have been successfully used for many years in the field of dentistry. However, metal alloys can undergo corrosion and can cause allergies. Moreover, metal-free restorations hold a key place in today’s dental practice, this aspect mostly being related to the increased demand for aesthetics. PEEK (polyether ether ketone) material—a high performance polymer—demonstrated superior mechanical properties, with different applications in dentistry, such as: implantology (implant abutments, temporary abutments, customized healing abutments, healing caps, implants), prosthodontics (single crowns and fixed partial dentures—interim or definitive, removable partial dentures, maxillofacial prosthodontics, occlusal splints), intra-radicular posts, and orthodontics. PEEK (polyether ether ketone) infrastructures can be veneered with composite resin, as an accepted solution for implant-supported fixed dental prostheses designated for patients with metal allergies. Moreover, PEEK (polyether ether ketone) material can be considered as a valuable alternative to titanium or zirconia, due to its high-quality mechanical properties. However, in prosthodontics, PEEK (polyether ether ketone) is used both as a long-term-provisional and a definitive material.

**Figure 2:**
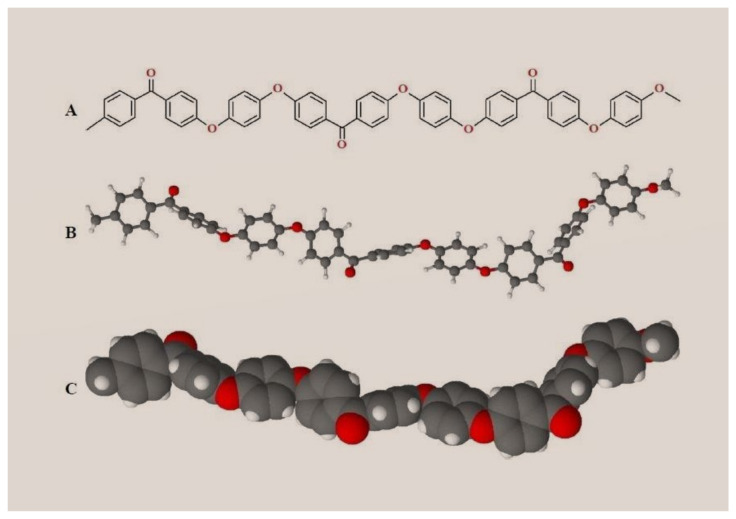
PEEK (polyether ether ketone) molecular structure, (**A**) phenylene rings (aryl), (**B**) oxygen bridges (R-O-R), (**C**) carbonyl groups (R-CO-R).

Polyaryl ether ketone (PAEK) is a semi-crystalline high-performance thermoplastic polymer. Its molecular backbone is built by phenylene rings (aryl), oxygen bridges (R-O-R), and carbonyl groups (R-CO-R) [79]. The PAEK (polyaryl ether ketone) family includes several members according to the presence of different sequences and ratios of aryl, R-O-R and R-CO-R groups in the macromolecular skeleton [80]. PEEK’s molecular structure is represented in Figure 2.

Due to its esthetic properties, PEEK (polyether ether ketone) attracts more and more attention in the continuously developing field of dental dentistry [79,81]. In 2014, Borgonovo et al. anticipated that PEEK (polyether ether ketone) would become suitable for digital dentistry [82]. Certain aspects regarding the correlation between PEEK (polyether ether ketone)’s chemical structure and the oral cavity conditions have already been demonstrated to be favorable: the ether groups (R-O-R) ensure structural flexibility, ketone groups (R-CO-R) give rigidity, while the phenylene groups are chemically unreactive. Consequently, the three functional groups give PEEK (polyether ether ketone) an excellent resistance to chemical attack, good processability, toughness, and high strength [3,81,83,84,85,86]. It has been pointed out that PEEK (polyether ether ketone) has a good resistance to hydrolysis [80,87]. PEEK (polyether ether ketone)’s special chemical structure favors both the chemical stability (illustrated by the resistance to most substances apart from concentrated sulfuric acid or a mixture of sulfuric acid and hydrogen peroxide—which can be used to roughen the PEEK (polyether ether ketone) surface), and its special physical properties as well (stability at high temperatures being very important for the sterilization processes) [88]. The study of Liebermann et al., which included an in vitro physicomechanical characterization of ceramic filled (20%) PEEK (polyether ether ketone), and certain esthetic dental CAD/CAM polymers (a hybrid material for definitive prosthetic restorations, a CAD/CAM nanohybrid composite for definitive prosthetic restorations, a CAD/CAM PMMA (poly (methyl methacrylate))-based material for temporary prosthetic restorations, and a bis-acrylate composite with nanofillers for temporary prosthetic restorations) showed that PEEK (polyether ether ketone) has the lowest solubility and water absorption values [88].

It has been highlighted recently that PEEK (polyether ether ketone) macromolecules display fatigue and abrasion resistance, good shock absorption, and, most importantly, high stability in the oral cavity without any physicochemical changes [3,81,86]. Moreover, PEEK (polyether ether ketone) has a closely related elasticity modulus value (approx. 4 GPa) with human bone (approx. 14 GPa), representing an important advantage of this polymer compared to conventional titanium/zirconium-based implants. Differences in elastic modulus between bone and diverse biomaterials could enhance the risk of bone mechanical overloading, triggering bone remodeling [80]. This polymer has also revealed tensile properties (approx. 80 MPa) similar to dentine (approx. 104 MPa) and natural enamel (approx. 68 MPa) when compared to zirconia (approx. 210 GPa, 550 MPa) and titanium alloy (approx. 110 GPa, 1200 MPa) [3,79]. Recent studies revealed that PEEK (polyether ether ketone) can be combined with other dental materials, such as ceramics [81,89,90]. It has been demonstrated that due to its special chemical and physical properties, PEEK (polyether ether ketone) can be successfully processed via computer-aided design and computer-aided manufacturing technology (CAD/CAM) [81,86].

Besides its unique combination of chemical and physical properties, one of the most important advantages of PEEK (polyether ether ketone) is represented by its potential to show good biocompatibility towards oral tissues [80,87,91]. Recently, the study presented by Peng et al. confirmed that, under the same culture conditions, PEEK (polyether ether ketone) incubated human oral fibroblasts showed cell adhesion effectiveness, metabolic activity, and pro-inflammatory responses similar to titanium alloy incubated fibroblasts [92]. Cell adhesion represents a vital molecular process for fibroblasts in order to survive on a material surface; only after the completion of this molecular event can other important cellular phenomena—such as cell proliferation, differentiation, diffusion and migration—take place [92,93,94,95]. All these cellular phenomena are strongly correlated with collagen secretion, tissue regeneration, and wound healing. The substrate roughness, mechanical properties, surface energy, and wettability play key roles in the way cell adhesion occurs [92,93,94,95]. Both PAEK and PEEK (polyether ether ketone) macromolecules have higher hydrophobicity and lower surface energy compared to ceramic or metallic materials, due to the reduced number of polar functional groups on their surface [92,93,94,95]. Peng et al. reported that in contrast to PEKK, PEEK (polyether ether ketone) (which has more ether groups) had a higher surface energy and lower contact angle. Hydrogen bonds cannot form between ether molecules. However, in the ether groups, there are a lot of non-bonding electron pairs on the oxygen atom, able to form hydrogen bonds with -OH or N-H [92,93,94,95]. Consequently, PEEK (polyether ether ketone) has a relatively high polarity. PEEK (polyether ether ketone), benefiting from a higher polarity when compared to PEKK, is more accessible for cells to adhere via specific cell membrane receptors, such as integrin, or through attachment proteins (i.e., fibronectin, collagen) [92,93,94,95].

However, in the context of dental applications, one of PEKK’s disadvantages is that it is considered bioinert, thus limiting the osseointegration process, essential for the long-term clinical success of dental implants [96]. PEEK (polyether ether ketone) polymer could exhibit limited inherent osteoconductive properties when compared to titanium, leading to a negative impact on the osseointegration process [96]. In order to improve PEEK (polyether ether ketone)’s biological properties, different types of bioactive compound have been studied [97]. Recently, Dong et al. increased the osseogenicity of PEEK (polyether ether ketone) implants by the incorporation of a multifunctional micro-/nanostructured surface composed of hydroxyapatite and nickel hydroxide [96]. Montaño-Machado et al. loaded the PEEK (polyether ether ketone) macromolecules with ZnO (zinc oxide) nanoparticles in order to improve material–cell molecular interactions and to induce antibacterial properties [98]. Further studies are needed to assess the long-term performance of PEEK (polyether ether ketone) material so that it can be safely recommended and used as a valuable alternative to conventional prosthetic materials.

## 3. Definitive Prosthetic Materials Used for Obtaining Oral Implant-Supported Prostheses

### 3.1. Materials and Techniques Used for Fabrication of Definitive Implant-Supported Prostheses

Nowadays, dental clinicians can benefit from a wide range of definitive prosthetic materials, both conventional and modern ones, for the fabrication of implant-supported fixed prostheses (metal–ceramic prosthesis—including ceramic-veneered titanium; metal–resin prosthesis; monolithic zirconia prosthesis; ceramic-veneered zirconia prosthesis; lithium disilicate prosthesis; hybrid ceramics prosthesis). The selection of these materials for a specific clinical case is correlated with the implant-supported prosthesis design, number of implants, implant location (upper or lower jaw), type of connection, aesthetic requirements, masticatory force, static and dynamic occlusal scheme, and chewing pattern [99,100,101,102,103,104,105,106,107]. It is acknowledged that metal–ceramic fixed dental prostheses (cast metal infrastructure and veneering ceramic) exhibit a good long-term clinical survival rate, both in the anterior and posterior regions of dental arches [99]. Metal–ceramic restorations have for many years been considered the standard for implant-supported prostheses due to their adequate strength and acceptable esthetics. The implementation of CAD/CAM technology in dentistry and the increasing demand for esthetic restorations led to the development of zirconia-based restorations.

### 3.2. Zirconia—A Successful Definitive Implant-Supported Prostheses Material

Zirconia is a polycrystalline ceramic, which has excellent biomechanical properties; however, this material exhibits several disadvantages related to the veneering ceramic bond strength, the high fracture rates of veneering ceramic, and the possibility of its degradation in the oral cavity [101]. Despite this, ceramic-veneered zirconia has been successfully used for implant-supported prostheses as it presents better esthetics than metal–ceramic prostheses, very good mechanical behavior, and excellent biocompatibility. Moreover, monolithic zirconia prostheses have been reported to be more fracture-resistant, the incidence of ceramic chipping being eliminated [102].

In the clinical study conducted by Nejatidanesh et al. [103] (2020), 114 posterior implant-supported fixed dental prostheses (FDPs), including zirconia-based prostheses (52) and metal–ceramic prostheses (62), were evaluated in a 5-year follow up; the study included 114 patients with a mean age of 59 ± 8.4 years. The results showed that the soft tissue status was not affected by the type of restoration, except for the plaque index—which was more favorable for zirconia-based FDPs (*p* < 0.001). No significant difference was found between marginal bone loss corresponding to the two groups of prosthetic restorations (*p* = 0.30 mesial, *p* = 0.46 distal). The authors concluded that zirconia-based and metal–ceramic FDPs showed similar clinical performance.

In 2021, Shen et al. [102] presented a study conducted on 224 participants treated with 327 oral endosseous implants; the implants were restored with either metal–ceramic or monolithic zirconia single crowns in the posterior region of the dental arches, between 2012 and 2016. The authors assessed the clinical outcomes, including the plaque index, peri-implant probing depth, bleeding on probing, and the marginal bone level (that was recorded by using the panoramic radiographs obtained at implant placement, at second-stage surgery, and at the most recent follow-up visit). The mean follow-up time was 30.4 months; the registered cumulative survival rate of implants was 100%, with that of the prostheses being 99.1%. The metal–ceramic group’s registered plaque index was 0.46, which was significantly higher (*p* < 0.05) than the monolithic zirconia group’s registered plaque index (0.37). However, no significant differences (*p* > 0.05) were observed between the two groups in peri-implant probing depth and bleeding on probing. The registered marginal bone level was above the implant platform in both the metal–ceramic and monolithic zirconia groups. The marginal bone level changes registered for the metal–ceramic group were 0.31 mm in the healing period and 0.38 mm in the prosthetic loading period, while in the monolithic zirconia group, these were 0.25 mm in the healing period and 0.43 mm in the prosthetic loading period; no significant differences (*p* > 0.05) were observed between the two groups. The change in peri-implant bone level was comparable after prosthetic loading for metal–ceramic and monolithic zirconia single crowns, although monolithic zirconia was associated with reduced plaque. The results of this study provide clinical evidence for the excellent performance of implant-supported monolithic zirconia prostheses in the posterior region of dental arches. These findings join other relatively recent ones [104,105,106], which outlined that the prosthesis material showed little or no effect on the peripheral bone. These aspects need to be confirmed in further clinical studies.

In a review published in 2019 by Bagegni et al. [107] that included forty-one studies related to the restorative prosthetic material’s influence on implant and prosthetic survival of implant-supported fixed complete prostheses, for a mean follow-up period of more than 3 years, the authors pointed out that a statistically significant difference (*p* = 0.0337) was found between implant survival rates of the main restorative groups (metal–ceramic—97%; all-ceramic—99%; metal–resin—97%). The results of this study showed that the prosthetic survival rates were: metal–ceramic—95%; all-ceramic—97%; metal–resin—97%; with no statistically significant difference (*p* = 0.3796) between the groups. Chipping incidence rates were reported as follows: metal–ceramic—8%; all-ceramic—15%; metal–resin—22%. The authors concluded that the prosthetic material selection seems to have no clinically relevant influence on implant- and prosthetic survival rate in implant-supported fixed complete dentures.

**Figure 3:**
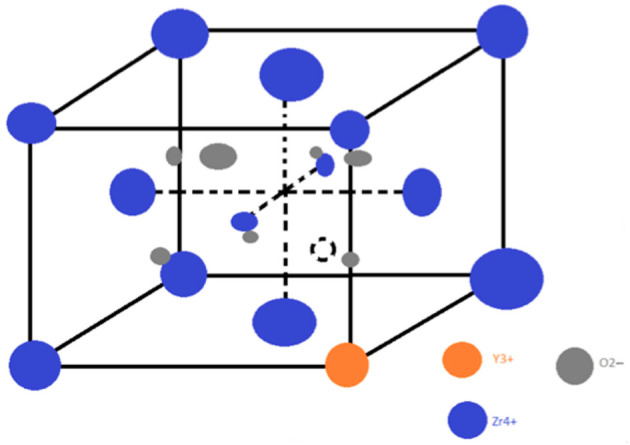
The yttria-stabilized zirconia structure.

Considering the above-mentioned elements regarding the implant- and prosthetic survival rate in implant-supported prostheses, aspects related to the biochemical interaction of zirconia with the oral environment will be also highlighted, as follows. Zirconia-based dental ceramics are chemically inert materials with no adverse effects on oral tissues. Three-mol percent yttria-stabilized tetragonal zirconia polycrystal (3Y-TZP), which is commercially available, represents one of the most commonly used type of zirconia [108,109,110]. Figure 3 represents the structure of yttria-stabilized zirconia.

Zirconia-based dental prostheses benefit from highly polished surfaces; consequently, their contact with the gingival tissues is favorable, playing an important role in the maintenance of the gingival architecture and allowing good cell adhesion and no adverse systemic reactions [111]. However, it has been reported that zirconia particles formed during degradation at low temperatures or resulting from the manufacturing processes may be released, triggering a local and oral inflammatory reaction [111]. Nevertheless, zirconia’s biocompatibility has been thoroughly evaluated overtime, and its high biocompatibility was demonstrated. Back in 1976, the study conducted by Styles et al. [112] highlighted the fact that zirconia did not induce cytotoxicity in soft tissues. More recent studies pointed out that no systemic or local adverse reactions produced by zirconia (no matter the tested structural form) have been reported, as presented in Table 1 [113,114,115,116,117,118,119,120,121].

The zirconia biocompatibility was evaluated in vitro by monitoring different cell culture interactions with the biomaterial [115,120]. In a recent study, conducted by Wei et al., murine pre-osteoblasts cells and human dental pulp stem cells were cultured on zirconia and titanium surfaces [121]. The cell viability and morphology were evaluated at 3, 12, and 24 h from seeding [121]. Intracellular ROS levels of both cell types were determined 24 h after seeding [121]. The zirconia samples revealed a significantly higher human dental pulp stem cell viability after 12 h from seeding (*p* < 0.05), compared to titanium samples [121]. Moreover, both cell types cultured on zirconia showed relatively higher mean ROS levels, 24 h after seeding, compared to titanium [121].

Intracellular ROS levels may be regarded as markers of the cellular status during the initial contact and adhesion on a material surface. ROS are known as subtle but very important regulators of cytoskeleton arrangement, cell-surface adhesion, and cell growth and spreading [122,123]. Moreover, the study of Vilas-Boas et al. highlighted that the low levels of intracellular ROS significantly delayed the cell adhesion and spreading processes. Consequently, Wei et al.’s results might indicate that the cells cultured on the zirconia surface were more active in cell adhesion and spreading [121]. However, further, more detailed investigations are needed in order to sustain intracellular ROS levels as markers of the cell adhesion process’ status.

Our scientific literature search has led to the acquisition of relevant data on biochemical oral interactions of prosthetic materials used on implant-supported restorations. Most of the this research’s findings are positive, favorable, highlighting the acknowledged qualities of the interim and definitive prosthetic materials that are nowadays used in oral implant therapy. However, the collected literature data also indicate that PMMA (poly (methyl methacrylate))-based materials used for obtaining interim implant-supported prosthesis raise certain problems concerning their biochemical interaction with the oral environment (i.e., elution of residual monomer, interaction of MMA (methyl methacrylate) monomers with human epithelial and pulp-cells, and influence of MMA (methyl methacrylate) on glutathione (GSH) or reactive oxygen species (ROS) levels). However, the introduction of modern bio-materials in clinical practice and the implementation of digital technology in dentistry (including subtractive technology and additive manufacturing) have led to outstanding progress over the last few years [124,125,126]. Recent studies pointed out that CAD/CAM technology and indirect fabrication methods allow the obtainment of interim prosthetic restorations with better biochemical oral responses when compared to the direct fabrication methods that require the usage of conventional polymers [126,127].

Besides the biochemical aspects related to prosthetic materials that were previously mentioned in this paper, modern scientific research includes other aspects of high interest. In this regard, antimicrobial prosthetic materials have been developed along with new advances in material science and engineering [128]. Nowadays, biomedical surfaces with integrated antifouling, and self-adaptive antimicrobial strategies, are intensively studied [128]. It is worth noting that the antibacterial properties of prosthetic materials used for obtaining implant-supported prostheses play a very important role in the longevity of dental implants [129,130,131,132,133,134,135,136]. The antibacterial capacity of natural polymers [129,130,131], the addition of antimicrobial agents in polymer matrix [129,132], antibacterial coatings of both dental implants and prosthetic materials [133,134], and the incorporation of metallic, ceramic, or polymeric antimicrobial nanoparticles [133,134,135,136] may prevent microbial colonization, infection, and subsequent oral implant failure [133]. The multifunctional antimicrobial materials not only fight against oral infections, but they can promote the efficacy of medical devices as well [128].

Moreover, it is estimated that, in the near future, personalized prosthetic biomaterials could be developed and improved, based on the important acquisition of data at the individual level (biomarkers), including at the salivary level [15,137]. Furthermore, the application of machine learning in material science will allow the acceleration of development in biomaterial manufacturing [138].

## 4. Conclusions

The biocompatibility and biomechanical properties of materials used in obtaining implant-supported prostheses represent important topics in modern scientific research. The practical relevance of the studies belonging to this field of dentistry consists of providing guidelines and helpful information for dental clinicians, in order to select the appropriate materials for each clinical case.

The present paper describes several favorable properties of PMMA (poly (methyl methacrylate)), a recognized interim prosthetic material. However, different aspects regarding the biochemical interaction of PMMA (poly (methyl methacrylate)) with the oral environment are presented (cytotoxicity, monomer release, influence on salivary redox status), which raise concerns about possible adverse oral effects caused by this material. These aspects become even more significant for dental implant therapy, which requires favorable soft and hard oral tissues reactions. The incorporation of NAC (*N*-acetyl cysteine) might be regarded as an effective strategy to obtain more biocompatible and clinically reliable PMMA (poly (methyl methacrylate))-based dental resins.

Relevant properties of PEEK (polyether ether ketone)—a high-performance polymer, which was more recently introduced on the dental market as a promising biomaterial—are also presented in this paper. PEEK (polyether ether ketone) can be used both as a long-term interim and a definitive prosthetic material due to its esthetics, suitability for digital manufacturing, physical properties, chemical stability, and favorable interaction with oral environment. Few long-term clinical studies are available on the use of PEEK (polyether ether ketone) in clinical dental practice; therefore, more scientific research is needed on the topic.

This paper also focuses on several prosthetic materials designated for obtaining definitive implant-supported prostheses. The long-term success of metal–ceramic implant-supported prostheses has already been demonstrated. On the other hand, zirconia-based prostheses (ceramic-veneered zirconia prostheses or monolithic zirconia ones) present better esthetics than metal–ceramic prostheses, very good mechanical behavior, and excellent biocompatibility. Recent scientific studies ascertain that zirconia, no matter the tested physical form, is regarded as a valuable biocompatible material. Relevant aspects regarding zirconia’s interaction with the oral environment were outlined in this paper, highlighting its very good biocompatibility in the mouth. Zirconia exhibits excellent biomechanical properties, but it has certain disadvantages related to the high fracture rates of veneering ceramics and the possibility of degradation in the oral environment. However, zirconia is highly recommended for obtaining definitive implant-supported prostheses.

Further research with standardized parameters for assessment of biochemical oral interaction of interim and definitive prosthetic materials used for obtaining implant-supported prosthesis and long-term follow-up studies on the survival rate and complications of implant-supported prosthesis are still required.

## Figures and Tables

**Table 1:** Examples of in vitro and in vivo zirconia biocompatibility studies.

Studied Cell Type and/or Tissue	Study Conclusions	Reference
Fibroblasts	Yttria-stabilized tetragonal zirconia polycrystals (3Y-TZPs) ceramic did not induce any mutagenic or cellular transforming effects.	[113]
Osteoblasts	Zirconia ceramics did not alter cell ploidy or the cell growth rate.	[114]
Macrophages	Zirconia ceramics particles induced macrophage apoptotic cell death, in vitro.	[115]
Fibroblasts; subcutaneous implant test	ZrO_2_/Al_2_O_3_ composite showed no cytotoxicity and no significant adverse effects in soft tissues.	[116]
Osteoblasts	Zirconia samples insured good levels of biocompatibility.	[117]
Osteoblasts	ZrO_2_, Al_2_O_3_, and PMMA (poly (methyl methacrylate)) particles triggered direct effects on osteoblasts. Cell responses depended on the particle type. ZrO_2_ effect on alkaline phosphatase activity was targeted to the matrix vesicles.	[118]
Bone and muscle; Fibroblasts	New zirconia implants illustrated good biocompatibility and mechanical properties.	[119]
Osteosarcoma-derived osteoblasts (SaOs-2); human gingival fibroblasts (HGF);monocytes (THP-1)	Zirconia particles affected the viability of SaOs-2 and HGF, but did not induce proinflammatory reactions in THP-1.	[120]
Human dental pulp stem cells; murine pre-osteoblasts	Zirconia as a potential dental implant material, illustrated similar or, even, better initial cellular responses versus titanium.	[121]

## Data Availability

The data are contained within the article.
